# Closed Loop Deep Brain Stimulation for PTSD, Addiction, and Disorders of Affective Facial Interpretation: Review and Discussion of Potential Biomarkers and Stimulation Paradigms

**DOI:** 10.3389/fnins.2018.00300

**Published:** 2018-05-04

**Authors:** Robert W. Bina, Jean-Phillipe Langevin

**Affiliations:** ^1^Division of Neurosurgery, Banner University Medical Center, Tucson, AZ, United States; ^2^Neurosurgery Service, VA Greater Los Angeles Healthcare System, Los Angeles, CA, United States; ^3^Department of Neurosurgery, University of California, Los Angeles, Los Angeles, CA, United States

**Keywords:** closed-Loop DBS, PTSD, addiction, facial recognition software, autism, schizophrenia, functional neurosurgery

## Abstract

The treatment of psychiatric diseases with Deep Brain Stimulation (DBS) is becoming more of a reality as studies proliferate the indications and targets for therapies. Opinions on the initial failures of DBS trials for some psychiatric diseases point to a certain lack of finesse in using an Open Loop DBS (OLDBS) system in these dynamic, cyclical pathologies. OLDBS delivers monomorphic input into dysfunctional brain circuits with modulation of that input via human interface at discrete time points with no interim modulation or adaptation to the changing circuit dynamics. Closed Loop DBS (CLDBS) promises dynamic, intrinsic circuit modulation based on individual physiologic biomarkers of dysfunction. Discussed here are several psychiatric diseases which may be amenable to CLDBS paradigms as the neurophysiologic dysfunction is stochastic and not static. Post-Traumatic Stress Disorder (PTSD) has several peripheral and central physiologic and neurologic changes preceding stereotyped hyper-activation behavioral responses. Biomarkers for CLDBS potentially include skin conductance changes indicating changes in the sympathetic nervous system, changes in serum and central neurotransmitter concentrations, and limbic circuit activation. Chemical dependency and addiction have been demonstrated to be improved with both ablation and DBS of the Nucleus Accumbens and as a serendipitous side effect of movement disorder treatment. Potential peripheral biomarkers are similar to those proposed for PTSD with possible use of environmental and geolocation based cues, peripheral signs of physiologic arousal, and individual changes in central circuit patterns. Non-substance addiction disorders have also been serendipitously treated in patients with OLDBS for movement disorders. As more is learned about these behavioral addictions, DBS targets and effectors will be identified. Finally, discussed is the use of facial recognition software to modulate activation of inappropriate responses for psychiatric diseases in which misinterpretation of social cues feature prominently. These include Autism Spectrum Disorder, PTSD, and Schizophrenia—all of which have a common feature of dysfunctional interpretation of facial affective clues. Technological advances and improvements in circuit-based, individual-specific, real-time adaptable modulation, forecast functional neurosurgery treatments for heretofore treatment-resistant behavioral diseases.

## Introduction

Closed loop deep brain stimulation (CLDBS) paradigms are heralded as the future of DBS systems. In our experience using open loop DBS (OLDBS) to treat psychiatric conditions, we encountered several limitations: (1) Relying on short-term clinical outcomes to identify optimal stimulation parameters is inadequate; (2) The continuous stimulation paradigm commonly used with DBS interferes with the normal function of the target structure impeding clinical improvement; (3) Monitoring the progression of the illness using clinical outcomes scores is insufficient to determine true network engagement and disease modification in the context of invasive neuromodulation. The closed loop systems can address those limitations. CLDBS systems promise circuit based learning and adaptation, improved system efficacy, increased safety, and reduced risk of tissue damage from prolonged stimulation. OLDBS, however, requires input through a human interface, unadaptable monomorphic stimulation between programming, habituation to stimuli, attenuation of response, and diminished efficacy over time (Santos et al., 2011; Richardson, 2012; Carron et al., 2013; Hosain et al., 2014; Hamilton et al., 2015; Sharma et al., 2016; Widge and Sahay, 2016; Widge et al., 2016, 2017).

Closed loop systems have demonstrated superiority over open loop systems in a variety of animal and human trials. Epilepsy treatments using closed loop methods in humans (Kossoff et al., 2004; Anderson et al., 2008; Morrell, 2011; Little et al., 2013; Eggleston et al., 2014; Heck et al., 2014; Bergey et al., 2015; Vonck and Boon, 2015; Geller et al., 2017; Jobst et al., 2017) show better seizure control when compared to OLDBS systems and movement disorder CLDBS trials in non-human primates (Rosin et al., 2011) and humans (Little et al., 2013) have demonstrated improvements in symptom control. More reports of CLDBS (previously confined to computational models; Holt et al., 2016; Liu et al., 2016) in humans are beginning to appear in the literature (Khobragade et al., 2015; Arlotti et al., 2016).

The single most relevant drawback to implementation of CLDBS paradigms is the identification of biomarkers for any given disease: the difficulties lie in detecting relevant changes in firing patterns in the background noise of the brain; in detecting and transmitting real-time alterations in neurotransmitter concentrations in relevant locations; and in determining which physical biomarkers are pathologic and which are not (Carron et al., 2013; Hosain et al., 2014). The ideal biomarker is one that can be followed in real-time and that correlates with symptoms and network activation. Work continues in CLDBS for movement disorder using a wide variety of parameters extracted from peripheral biomarkers of disease to predict tremor (Khobragade et al., 2015; Widge and Sahay, 2016; Widge et al., 2017) and in psychiatric disease through use of invasive monitoring of local field potentials and stimuli in brain regions of interest in epilepsy patients (Widge and Sahay, 2016; Widge et al., 2017). In this report, we propose the hypothetical application of CLDBS to Post-Traumatic Stress Disorder (PTSD), addiction disorders, and disorders affecting social skills.

### Methods

MEDLINE/Pubmed were searched on July 12, 2017 and on March 28, 2018. No date limits were applied. The following terms were used: Closed Loop Deep Brain Stimulation, Deep Brain Stimulation PTSD, Deep Brain Stimulation Chemical Dependency, Deep Brain Stimulation Alcohol, Deep Brain Stimulation Gambling, Neurobiology Addiction, PTSD Biomarkers, Facial Recognition Software, Responsive Neurostimulation, Facial Recognition Autism, Facial Recognition PTSD, Facial Recognition Schizophrenia. All articles returned were in English. Articles which were assessed were specifically relevant to the aforementioned search topics.

**Figure 1:**
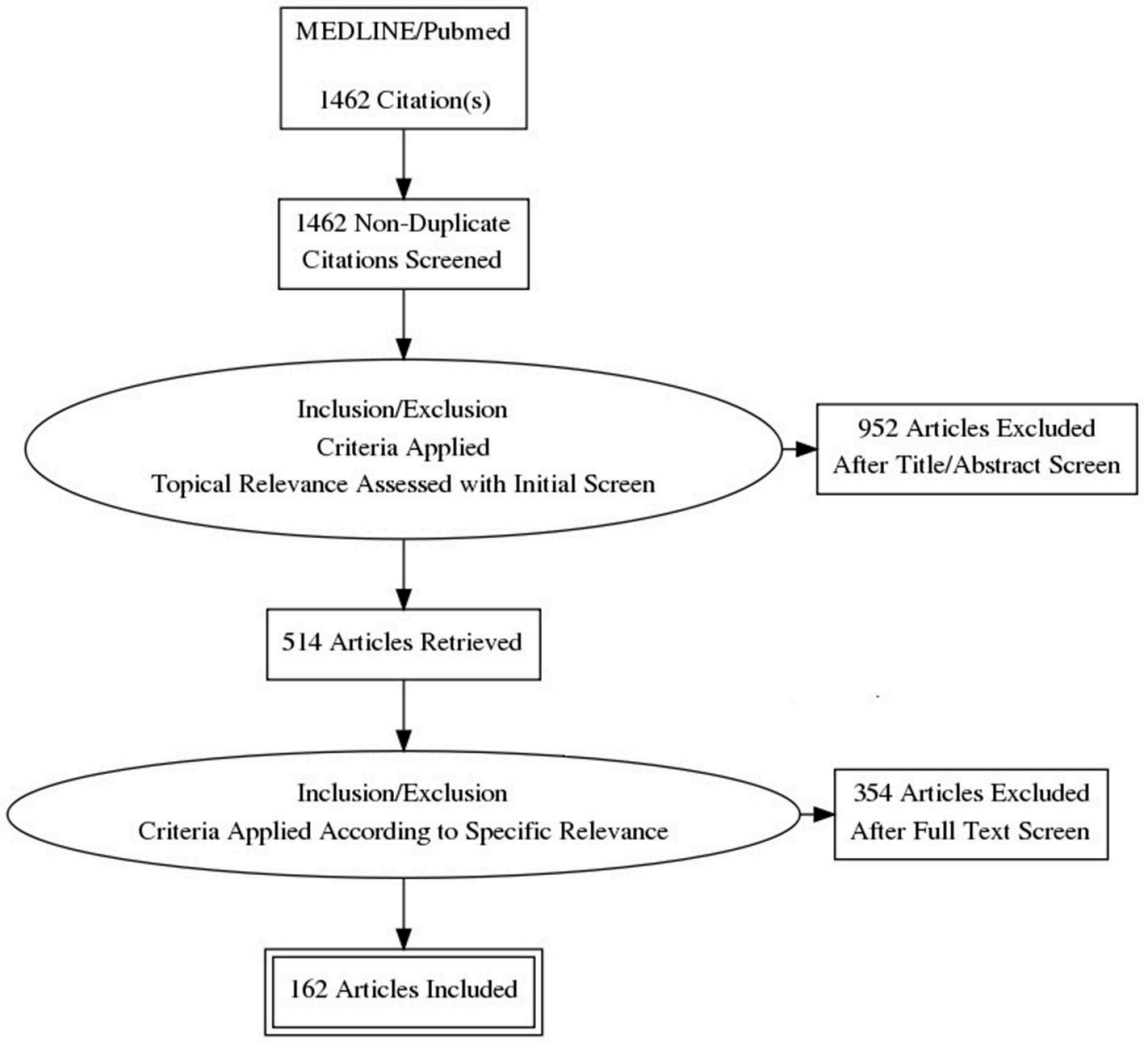
PRISMA flowchart.

A total of 1,460 articles were screened, 514 were assessed, and 162 were classified and included for the construction of the manuscript (Figure 1): 14 publications were classified under Neurobiology of Addiction and/or PTSD; 15 under Responsive Neurostimulation; 15 under CLDBS; 40 under PTSD Biomarkers and Facial Afective Procesing; 27 under Behavioral Addiction; 27 under Chemical Addiction; 14 under Facial Affective Processing Schizophrenia or Autism; and 5 under Facial Expression Analysis.

## Closed loop deep brain stimulation for post-traumatic stress disorder

PTSD is categorized as a Trauma and Stress Related Disorder in the American Psychiatric Association Diagnostic and Statistical Manual (DSM) of Mental Disorders V (American Psychiatric Association, 2013). OLDBS targets for PTSD patients have been proposed with lead placement in the amgydala (Langevin et al., 2010; Langevin, 2012; Stidd et al., 2013; Koek et al., 2014) with a single case report of improved symptomatology (Langevin et al., 2016) after stimulation. OLDBS has also been demonstrated to be effective in reducing stereotyped behaviors in a rat model of PTSD and was superior to medical treatment (Stidd et al., 2013).

**Figure 2:**
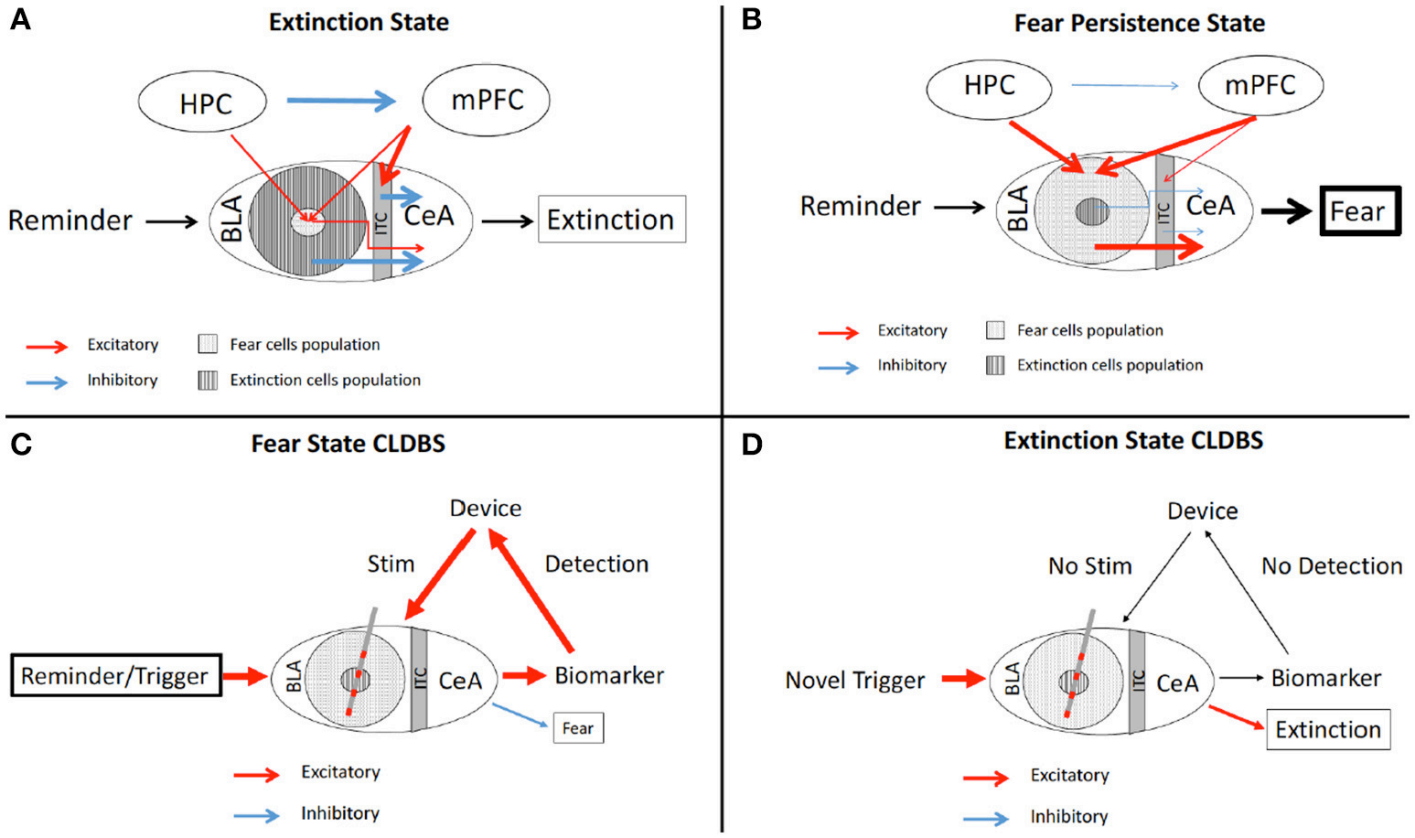
Schematic of fear circuitry and CLDBS for PTSD. The fear extinction **(A)** and persistence **(B)** circuits with output behaviors. In the Extinction state **(A)**, the BLA extinction cells and the ITC cells inhibit the CeA cells, resulting in fear extinction. The HPC inhibits the mPFC resulting in stimluation of the ITC cells and attenuated stimulation of the fear population cells in the BLA. **(B)** In the Persistent Fear state, the fear cells and extinction cell proprotions are reversed with fear cells predominating. The HPC and mPFC hyperstimluate the fear cell popualtions which then hyperstimluate the CeA, resulting in persistent fear behavior. **(C)** With placement of a CLDBS electrode in the BLA, some trigger generates an increase in a biomarker (please see text for discussion of biomarkers), which the device interprets and delivers a stimulus to the BLA, thereby inhibiting/attenuating the persistent fear response. **(D)** Over time, the reminder/trigger does not activate the biomarker and the device does not deliver a stimulus, allowing for the normal fear extinction to take place. BLA, basolateral amygdala; CeA, central nucleus; HPC, hippocampus; ITC, intercalated cells; mPFC, medial prefrontal cortex.

The amygdala's function in PTSD is known to be disordered and the fear, panic, and sympathetic response to the trigger stimulus is significantly out of proportion (Cisler et al., 2015). Furthermore, failure of fear extinction is a core component of PTSD. Fear extinction and fear consolidation are both regulated by the same network. At the center of this network, we find “extinction” cells and “fear encoding/consolidation” cells in the basolateral nucleus of the amygdala (BLn). These neurons mediate their respective role through their interaction with the medial prefrontal cortex (Lüthi and Luscher, 2014) (mPFC). The relative volume of active fear cells compared to active extinction cells in the BLn determines the ultimate outcome of the incoming trauma reminder. In the context of fear extinction, the BLn can be conceptualized as an emotional receptive field existing in two possible states: an extinction state and a consolidation state. The specific state of the BLn is determined by the influence from other nodes in the network such as the hippocampus (memory) and the mPFC (learning, context) (Figures 2A,B).

Several possible biomarkers can be considered for a hypothetical closed loop system. For instance one could consider: physical changes which come about due to hyper-activation of the sympathetic drive—pupillary dilatation, skin conductance changes, and piloerection (Lewine et al., 1997; Orr and Roth, 2000; Dierks et al., 2007; Pole, 2007; Pitman et al., 2012; Schmidt et al., 2013; Kang et al., 2015); intensified startle responses as measured by decreased reflex latency and degree of response (Butler et al., 1990; Schmidt et al., 2013); dysfunctional hypothalamic-pituitary-adrenal axis endocrine changes, such as changes in concentrations of corticotropin releasing hormone (Bremner et al., 1997; Baker et al., 1999) or other endocrine markers, such as oxytocin and vasopressin (Neumann and Landgraf, 2012; Olff, 2012; Schmidt et al., 2013; Frijling et al., 2015; Kang et al., 2015; Nishi et al., 2015); changes in neurotransmitters centrally and peripherally in serum (Hamner and Diamond, 1993; Southwick et al., 1993, 1999; Glover et al., 2003; Dierks et al., 2007; Strawn and Geracioti, 2008; Golub et al., 2011; Herrmann et al., 2012; Schmidt et al., 2013; Kang et al., 2015); and conceivably changes in firing patterns in the limbic circuitry which herald the fear generator (Wolf and Herringa, 2016).

Peripheral evidence of the activation of the sympathetic nervous in PTSD patients includes changes in electrodermal conductance and increased cardiac reactivity—elevated heart rate and blood pressure (Brende, 1982; Bryant et al., 1995; Blechert et al., 2007; Kozarić-Kovacić et al., 2010). Devices already exist that measure both electrodermal changes and ECG. Fletcher et al published a report in 2011 for a device that combines measurements of these two peripheral biomarkers of autonomic activation. Their device communicates to a handheld smartphone app and presents calming cognitive behavioral therapy messages to the wearer (Fletcher et al., 2011). This system could be adapted to DBS. When the peripheral sensors detect autonomic activation, the circuit could be activated. The effector leads, placed in the amgydala (Langevin et al., 2010, 2016; Shin and Liberzon, 2010; Langevin, 2012; Stidd et al., 2013; Koek et al., 2016), could then increase stimulation to attenuate hyper-activation responses (Figures 2C,D).

The difficulty inherent to using peripheral markers of activation lies in the low fidelity of autonomic responses—any major emotional change activating the sympathetic drive could be interpreted as pathological. The goal is not to attenuate every extreme deviation from normal physiology—and though these particular peripheral inputs may be the least invasive—they are blunt instruments and may not avail the most refined input. Epilepsy systems which monitor cardiac rates as a marker for impending seizure activity are widely in use (Eggleston et al., 2014) and are refined to deliver stimuli when cardiac reactivity matches patterns consistent with predetermined pre-ictal states.

Endovascularly placed chemosensors (Langevin et al., 2010), similar to microdialysis sensors in animal experiments for addiction (Pettit and Justice, 1991) could be placed to take real-time serum measurements of catecholamines, oxytocin, vasopressin, corticotropin releasing hormone, or other endocrinological markers. Stereotyped concentration changes, consistent with hyper-activation, could be communicated to the generator and analyzed there to activate the effector leads.

Perhaps the most refined input would be from sensor leads placed in the mPFC, VTA, or amygdala itself to measure local field potential changes consistent with hyperactivation and behavioral responses characteristic to the patient (Shin and Liberzon, 2010; Langevin, 2012). There is evidence from animal trials that cats exposed to foot shock, a common PTSD model, demonstrated a sustained increase in basolateral amygdala (BLA) neuronal firing and synchronicity. This pattern peaks 30–50 min after foot shock (Pelletier et al., 2005). Additionally, increases in BLA local field potential (LFP) changes were observed in rodents. The differences in LFP power was greatest in frequencies in the gamma range (25–40 Hz) between the conditioned stimulus (CS) paired with foot shock and without—CS+ and CS– (Fenton et al., 2013). Taken together, these results suggest the presence of a sustained increase in BLA LFP power in the gamma range after a trauma reminder in PTSD patients.

Using this input paradigm does require further scientific refinement of the circuitry and LFP characteristics as well as invasive placement of sensors. It does potentially provide the most accurate method for sensing relevant changes in the abnormal fear response.

Other potential biomarker modalities—peripheral measurements of autonomic activation and peripheral chemosensors—are the end products of circuit dysfunction. Measuring these peripheral biomarkers is less invasive than measuring central biomarkers of circuit dysfunction (e.g., changes in local field potentials) which could necessitate leads placed in the brain. In addition to allowing for input in a CLDBS system, using peripheral biomarkers correlated to disease symptoms could help the clinician follow the progression of the disease, therefore providing critical information even when the CLDBS stimulation may not provide significant clinical benefit.

Responsive neurostimulation for epilepsy (RNS, Neuropace) is a proprietary system used not only to treat medically refractory epilepsy (Fountas and Smith, 2007; Morrell, 2011; Sprengers et al., 2017) but also provides data to assist with tracking responses to stimulation, since the device provides an accurate measure of seizure frequency and distribution over time. In the case of highly unstable conditions, such as treatment-resistant PTSD, fluctuations with periods of exacerbation are inherent in the natural history of the disease. In these cases, a biomarker that tracks disease fluctuation could predict the occurrence of an exacerbation prior to onset and alert the clinician to increase supportive care to reduce the risks of suicide or other drastic behavior. Power spectral density analysis to evaluate for fluctuations in specific frequency ranges in the post-treatment phase compared to pre-treatment phase has proven useful to identify possible disease state biomarkers using responsive neurostimulation in other conditions such as Tourette's syndrome (Maling et al., 2012) and may be useful in PTSD. The Neuropace algorithms are proprietary, therefore, the use of responsive neurostimulation to treat PTSD may require development and refinement of published responsive closed loop systems (Peters et al., 2001; Kossoff et al., 2004; Osorio et al., 2005; Gordon et al., 2017) and algorithims (Osorio et al., 2001; Raghunathan et al., 2009; Sandler et al., 2018) for epilepsy. Nonetheless, systems such as Neuropace RNS or Medtronic RC+S offer research package that allow the extraction of electrophysiological data from the device for research purpose using Matlab or similar data analysis software. In this context, the devices offer an opportunity to obtain chronic recordings from relevant cerebral areas. In particular, those recordings can be obtained during symptomatic phases and compared to resting state potentially yielding relevant symptoms biomarkers. The recordings obtained in this manner can be analyzed using a broad array of paradigms (frequency, amplitude, phase coherence, line length, etc.) without being confined to specific hypotheses.

## Closed loop deep brain stimulation for addiction: chemical dependency

Historically, controversial ablations of the NAc were performed for opiate addicted patients with reported cessation of addictive use (Orellana, 2002; Gao et al., 2003). DBS as a potential treatment for addictive chemical dependency is a serendipitous side-effect of DBS for movement disorders. The first report of DBS addressing addictive chemical dependency was published in 2007 in which a patient treated for his severe anxiety and depression was treated with bilateral DBS of the Nucleus Accumbens (NAc). The patient had sustained, unaided reduction of alcohol consumption with abstinence prevailing. The report also details that the targeted symptoms of anxiety and depression were not much improved (Kuhn et al., 2007). Since that initial report, there have been several case reports and a limited number of case series specifically using DBS to target addictive chemical dependency [alcohol (Müller et al., 2009, 2013, 2016; Voges et al., 2013), nicotine (Kuhn et al., 2009), and heroin (Zhou et al., 2011)]. There are also many animal studies of addictive chemical dependency (Luigjes et al., 2012; Pierce and Vassoler, 2013) in which cocaine (Vassoler et al., 2008, 2013; Friedman et al., 2010; Rouaud et al., 2010), alcohol (Knapp et al., 2009; Henderson et al., 2010), heroin (Guo et al., 2013), morphine (Liu et al., 2008; Martínez-Rivera et al., 2016), and sucrose (Friedman et al., 2011) were used as primary use drugs with complete cessation of use or significantly reduced ingestion.

A recent case series using bilateral NAc DBS for alcohol use was published with promising results. Five patients with chronic alcohol addiction were electively implanted with electrodes and monitored for an average of 38 months post-operatively. All five patients reported considerably decreased cravings for alcohol and two of the five patients remained abstinent for the follow-up period (Voges et al., 2013).

The environmental cues for substance abuse are varied and abundant. Several simple measures are apparent. Global Positioning System (GPS) location cues or visual drug/drinking cues (paraphernalia, drug, or alcohol itself) could be interpreted by a peripheral sensor and an abortive stimulus could be delivered into a CLDBS circuit, attenuating the initial reward response pathway activation. Also, peripheral physiologic signals of autonomic activation, similar to those discussed above in PTSD, could trigger an abortive stimulus.

**Figure 3:**
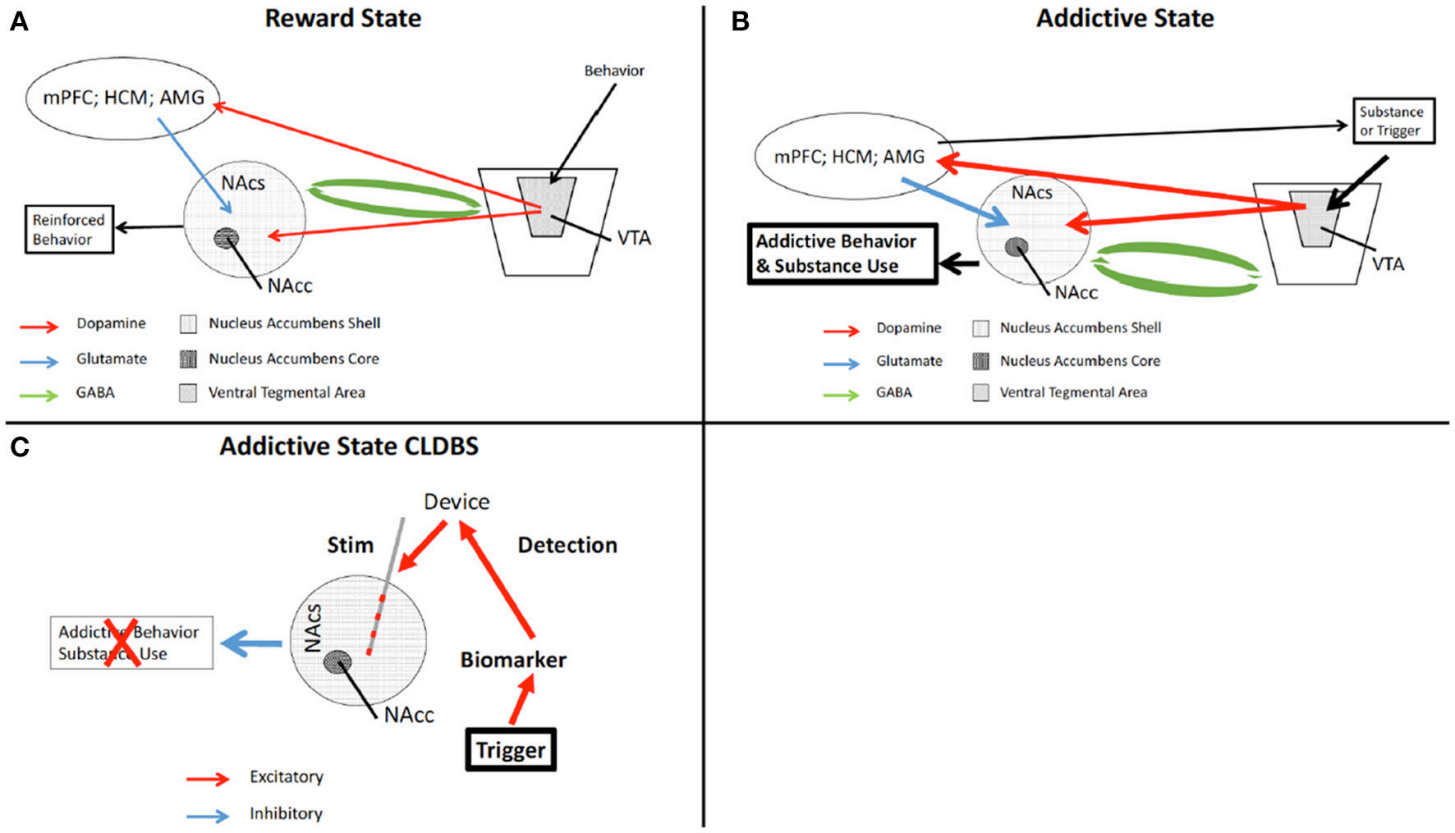
Schematic of reward circuitry and CLDBS for addiction. In the normal state, a behavior is performed which ultimately is determined to be rewarding. **(A)** The behavior triggers release of DA from the VTA which synapses on the NAcs, and the mPFC, HPC, and AMG, presented here as a single node for simplicity. The mPFC, HPC, and AMG then synapse onto NAcs neurons releasing excitatory glutamate. The NAcs and the VTA are reciprocally connected with inhibitory GABA neurons. The end result is a reinforced rewarding behavior. **(B)** When an addictive substance or behavior is introduced, reinforced by input from the mPFC. HPC, and AMG, the VTA is hyperactivated, increasing DA release in the NAcs. This also results in increase DA in the other nodes, increasing glutamate in the NAcs, with output being further motivation for increased substance use or addictive behavior (n.b., this is a simplification of this circuitry). **(C)** placement of a CLDBS electrode into the NAcs, some trigger generates an increase in a biomarker, which the device interprets and delivers a stimulus into the NAcs, thereby attenuating the motivational drive for the addictive behavior or substance use. AMG, amygdala; DA, dopamine; NAcs, nucleus accumbens shell; NAcc, nucleus accumbens core; VTA, ventral tegmental area.

The environmental, emotional, and psychological triggers for the phenomenon of craving in substance abuse in humans are varied, individualized, and difficult to interpret. What may trigger one person to compulsively seek their substance of choice may attenuate that compulsion in another. What is known is that the dopaminergic mesolimbic circuitry is intimately involved with the ritualized behavior leading up to actual substance use (Koob and Volkow, 2010; Volkow et al., 2012). Also known are nodes within that circuit which are primarily involved in the affected behavior—such as the NAc and Ventral Tegmental Area (VTA) (Koob and Volkow, 2010; Luigjes et al., 2012; Volkow et al., 2012; Wang et al., 2014) (Figures 3A,B).

When an individual is aroused and in the compulsive state of drug seeking, the changes in peripheral physiology are similar to the hyperarousal in PTSD. Peripheral sensors of autonomic activation—skin conductance and cardiac reactivity—could be similarly used to signify peripheral arousal. The CLDBS could then deliver an attenuating stimulus into the NAc, thereby aborting the dopaminergic surge preceding the behavior of addictive chemical use (Figure 2C). The benefit of such a paradigm is that the physiologic signs of arousal/anxiety precede the actual drug use (Koob and Volkow, 2010; Volkow et al., 2012). Again, as mentioned above, refinement of the input would be necessary to prevent indiscriminate attenuation of all pleasurable activities and all physiologic arousal. The goal is not anhedonia.

With global positioning software and person specific location targeting ability, CLDBS could utilize GPS position locators. Sensors could be linked with mapping software indicating an individual's physical place at a bar, at predetermined addresses (dealer, family, etc), or other places associated with substance use. Facial recognition software could also be used to identify particular persons who are associated with substance use for a patient; certain faces could be recognized via specialized contact lenses with facial recognition software capabilities (see discussion below of facial recognition software).

The higher cortical functions that trigger the dopaminergic surge ultimately end in the NAc but the hub of activity of the mesolimbic system is the VTA (Figures 3A,B). The dopaminergic medium spiny neurons in the VTA are reciprocally connected to the various nodes of the mesolimbic circuit (Nestler, 2005). The VTA is the control center and the NAc is the end effector. Sensors placed in the VTA could measure local field potentials. When the VTA is activated, dopamine is released in the various nodes of the mesolimbic circuit (Adinoff, 2004). The most robust response associated with addictive substance abuse is in the NAc. Chemosensors in the NAc could be used to detect local increases of dopamine. As mentioned previously, fine tuning of the dopaminergic changes would be necessary, as anhedonia is not the desired result, as was seen in 12.1% of patients with nucleus accumbens ablations for opiate addiction (Li et al., 2013). One of the potential side-effects of the OLDBS systems is alteration of the normal function of the target structure: one of the goals of CLDBS is to prevent a pervasive functional interruption and to provide a more specific signal normalization.

Once CLDBS leads are placed, as part of the system learning, a given patient's specific triggers could be examined for neural firing patterns preceding behaviors associated with addictive chemical use. These exposures can be performed in a controlled environment such as the clinic or a laboratory where the patient is presented cues of addiction while LFPs are recorded for evoked potentials. An alternate method is to use the on-demand recording feature of the device in real-life situations. In this paradigm, the patient activates the recording in the presence of specific relevant symptoms to the condition while in their regular environment. Device-based neural pattern recognition could then be programmed and stimulation modulation would follow, delivering appropriate stimuli in situations when the patient is more likely to use or drink. The methodology of obtaining, encoding, and interpreting these signals, both the environmental and physiologic, are conceivable and, at least in part, already available in commercially available devices such as the RNS^TM^ epilepsy system. As we mentioned in previous sections, the signals can be exported from the device to allow further analysis in Matlab. It is even conceivable that a sensor would be able to interpret visual cues and patterns specific to the individual.

## Closed loop deep brain stimulation for addiction: behavioral

Non-substance addictive behaviors, are becoming increasingly recognized as legitimate disorders (Potenza, 2015; Yau and Potenza, 2015; Banz et al., 2016; Gendreau and Potenza, 2016; Kraus et al., 2016; Mann et al., 2016; Fauth-Bühler et al., 2017) and the DSM V has included gambling with “Substance-Related and Addictive Disorders” (American Psychiatric Association, 2013). Within the field of psychotherapy and addiction treatment, stereotyped behavior patterns which display some obsessive-compulsive traits, distinct from obsessive-compulsive disorder, are referred to as behavioral addictions. These include, among others, gambling, on-line gaming, internet use, sex, shopping, exercise, and over-eating (Grant et al., 2010; Potenza, 2015; Mann et al., 2016). The DSM V concluded that there is inadequate scientific evidence to classify these behaviors as mental health disorders, with the exception of pathological gambling (American Psychiatric Association, 2013; Yau and Potenza, 2015).

It has been long known that patients with movement disorders can infrequently develop hypersexual (Doshi and Bhargava, 2008), overspending, overeating (Zahodne et al., 2011), and pathological gambling (Voon et al., 2006; Smeding et al., 2007; Santangelo et al., 2013) behaviors (Weintraub et al., 2010; Broen et al., 2011; Ramirez-Zamora et al., 2016; Kasemsuk et al., 2017) associated with the use of dopaminergic medications. These individuals have no prior history of these behaviors. These aberrant behaviors have been treated with medication changes (Weintraub et al., 2010; Lulé et al., 2012; Santangelo et al., 2013; Ramirez-Zamora et al., 2016) or with DBS in the bilateral subthalamic nuclei (STN) (Eusebio et al., 2013; Kasemsuk et al., 2017; Merola et al., 2017) which can result in the reduction or cessation of the problematic, presumably dopaminergic-driven behavior (Lim et al., 2009).

Specifically, compulsive gambling has been treated by DBS in an OLDBS system and with concurrent medication changes with good clinical results (Bandini et al., 2007; Eusebio et al., 2013; Castrioto et al., 2015; Merola et al., 2017). If, as is the case with the wide variety of substance abuse disorders, the addictive behaviors and cravings preceding use can be aborted with DBS, then it stands to reason that the same is true for behavior addictions.

The circuitry in behavior addiction is the same as the circuitry of substance abuse (Grant et al., 2006, 2010; Karim and Chaudhri, 2012; Leeman and Potenza, 2013; Love et al., 2015; Banz et al., 2016) but the mechanisms involved are far more delicate as all of the circuitry adaptation is within physiologic range (Nestler, 2005) (Figures 3A,B). The difficulty in treating behavioral addictions lies in the fact that most of the addictive behaviors are tied to normal physiologically or culturally necessary behaviors (Olsen, 2011; Leeman and Potenza, 2013).

Ingestion of a potentially addictive substance is, at its start, completely voluntary and not at all necessary for physiological survival. Behavior addictions, however, are tied to functions which are intimately tied to survival or cultural function (Nestler, 2005; Leeman and Potenza, 2013)—food is necessary for life, sex for propagation of the species, etc. The challenge in treating these particular behaviors with a CLDBS system will lie in that the pathologies are subtle derivations from normal. Physiological functional MRI changes associated with pathological sexual behavior were found in the striatum (Kühn and Gallinat, 2014; Seok and Sohn, 2015; Gola et al., 2017) and amygdala (Voon et al., 2014) with significant physiologic and functional overlap with internet gaming, overeating, gambling, and substance abuse (Voon et al., 2014; Love et al., 2015; Noori et al., 2016). A CLDBS system for behavior addictions would likely utilize many of the same triggers as would a substance abuse system (Voon et al., 2014; Love et al., 2015; Noori et al., 2016)—environmental and physiologic cues would trigger an abortive stimulus (Figure 2C). Much more investigation is needed into the physiologic neurocircuitry of these disorders.

## Closed loop deep brain stimulation for facial emotional interpretation

**Figure 4:**
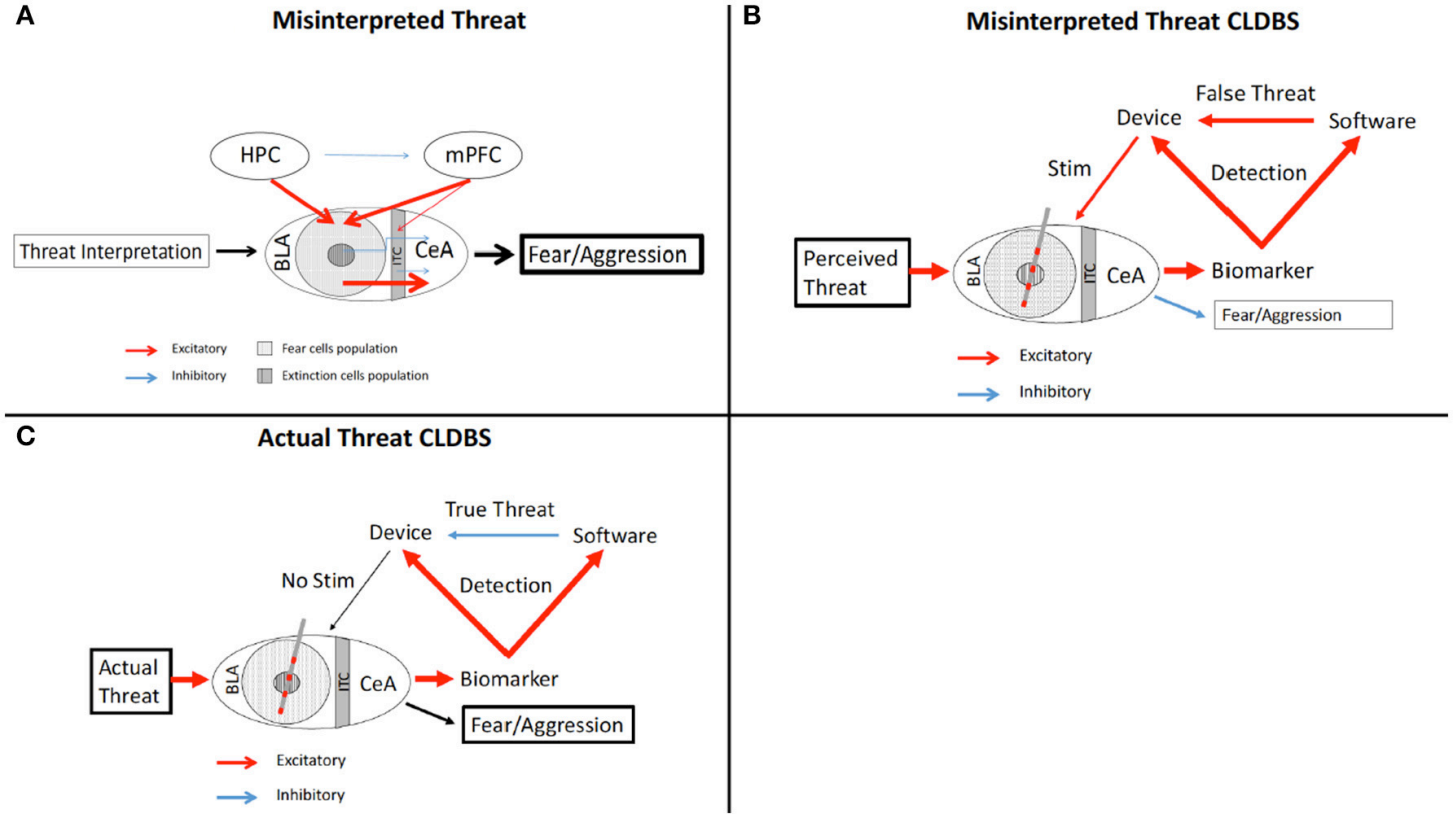
Schematic of fear circuitry and CLDBS for misinterpreted facial cues. **(A)** In the Misinterpreted Threat state, the HPC and mPFC inappropriately stimulate the fear cell populations which then stimulate the CeA, resulting in fearful and aggressive behavior. **(B,C)** With placement of a CLDBS electrode in the BLA, a threat generates an increase in a biomarker (please see text for discussion of biomarkers), which the device interprets. The biomarker also activates the facial recognition software to interpret the threat as false **(B)** or true **(C)**. If the threat activation is interpreted as false by the software **(B)** (facial recognition senses innocuous facial stimulus), the device is activated and an attenuating stimulus is delivered to the BLA, inhibiting a fear/aggressive response. If the threat activation is interpreted as true by the software **(C)** (facial recognition matches perceived threat), the device is inhibitied, no stimulus is delivered, and the fear/aggression response occurs in response to a true threat. BLA, basolateral amygdala; CeA, central nucleus; HPC, hippocampus; ITC, intercalated cells; mPFC, medial prefrontal cortex.

Several psychiatric disorders have a common feature of misinterpretation of visual and auditory cues. Specifically in Autistic Spectrum Disorder (ASD) (Uljarevic and Hamilton, 2013; Black et al., 2017), PTSD (Masten et al., 2008; Poljac et al., 2011; Shenk et al., 2013; Felmingham et al., 2014; Shu et al., 2014), and Schizophrenia (Ay et al., 2016; Belge et al., 2017; Krakowski and Czobor, 2017; Statucka and Walder, 2017), hypotheses suggest atypical processing of social cues transmitted through non-verbal communication. The deficits in these three disorders differ—atypical emotional processing in ASD, dysfunctional and misinterpretative processing in PTSD, and a global lack of facial processing in schizophrenia; however, all three conditions, regardless of underlying etiology, can result in misinterpretation facial cues—triggering inappropriate aggressive behavioral responses rather than a more appropriate response given an innocuous encounter (Figure 4A).

Recognition and interpretation of non-verbal communication is a complex developmental process that takes years to fully develop, starting in infancy and progressing through adulthood (Black et al., 2017). This process is generally accepted to be impaired in patients with ASD (Gay et al., 2013) and is stable across cultures (Uljarevic and Hamilton, 2013; Brewer et al., 2017) though not all experimental studies support this conclusion (Griffiths et al., 2017), fostering continued investigation. Significant research into the etiology of this impairment has been conducted with the use of eye tracking (van der Geest et al., 2002), EEG (Black et al., 2017; Malaia et al., 2017), and facial emotional recognition tasks (Gay et al., 2013; Uljarevic and Hamilton, 2013; Black et al., 2017; Malaia et al., 2017). The major atypia found in these studies is that patients with ASD misinterpret the basic facial features and varying degrees of the intensity of emotion related facial expressions (e.g., slightly irritated v. irate) (Griffiths et al., 2017). The attentional focus, as measured by eye tracking and other metrics, of ASD patients differs from typical patients (Hobson et al., 1988; van der Geest et al., 2002; Malaia et al., 2017) in that ASD patients focus on the mouth as opposed to the eyes during speech and communication. Also, EEG changes in brain regions associated with visual cues (P1, N170, and N300 patterns) and higher order processing varies in ASD (Dawson et al., 2004; Malaia et al., 2017).

Interpretation of facial affect has been demonstrated to be disordered in adults and adolescents with PTSD (Masten et al., 2008; Poljac et al., 2011; Shenk et al., 2013; Felmingham et al., 2014; Shu et al., 2014; Javdani et al., 2017). There also has been demonstration from EEG studies (Shu et al., 2014) and fMRI studies that various nodes in the fear circuit are dysfunctional in patients when presented with facial recognition tasks (Cisler et al., 2015; Wolf and Herringa, 2016). In schizophrenia, the misinterpretation of social cues judged through facial recognition (Ay et al., 2016; Krakowski and Czobor, 2017) lie not in a specific deficit of emotional processing, but in a more generalized disorganization of higher order visual processing (Jang et al., 2016; Mier et al., 2016; Belge et al., 2017) with a bias toward negative interpretation (Statucka and Walder, 2017).

CLDBS systems could interpret visual cues and facial features and monitor or modulate inappropriate responses. Facial recognition programming could, with physiologic and neurotransmitter sensors, function to interpret social cues more accurately and system output could deliver an abortive response to inappropriate activation of particular nodes in the circuit (Figures 4B,C).

The facial recognition software would interpret facial patterns according to typical responses. Inappropriate activation of the sympathetic system due to misinterpretation as threat of social cues by the patient could be then aborted as the sensing arm of the CLDBS system identifies mismatch of the response initiation and the facial recognition interpretation. These aberrations, interpreted by the device, could then signal for stimulus delivery to the effector node—the amgydala seems the likely target (Langevin, 2012; Langevin et al., 2016) (Figures 4B,C). Another possible node with relevance to interpretation of negative valence emotional cues is the lateral habenula, which has been implicated in aggressive behavior in animal models (Hikosaka, 2010; Golden et al., 2016).

Facial expression analysis software exists and is being increasingly refined (Chu et al., 2013, 2016; Cohn and De la Torre, 2015; Girard et al., 2015). These software analyze human facial expressions and accurately assess spontaneous facial action units through algorithmic pattern interpretation. As this software is tested in the lab and in the market, flaws in the algorithms are being discovered and addressed: precision and accuracy errors (White et al., 2015), lack of recognition of emotional subletly (Yitzhak et al., 2017), and extremely cumbersome coding (Girard et al., 2015). Prior to implementation of facial recognition into a CLDBS, the software precision and accuracy would need to be reliable, with the capability of recognizing not only obvious facial emotional expression, but subtleties as well, a task in which humans vastly outperform computers. The technological challenges that need to be resolved prior to integration into a CLDBS system are great, but progress is steady. The system may not require perfection prior to implementation, however. Although the risks related to delivering a percentage of inaccurate stimulation is unknown (i.e., stimulating for the wrong facial expression) it could still represent an improvement over chronic continuous DBS.

Additionally, cortical EEG implants placed over the parietal and temporal lobes monitoring the P1 and N170 patterns could detect aberrations which are consistent with scalp EEG aberrations noted in trials of ASD patients misinterpreting facial cues (Black et al., 2017; Malaia et al., 2017). If a cortical EEG pattern is mismatched to facial recognition software interpretation in conjunction with local field potential changes in the amygdala, an abortive stimulus could then be delivered, thereby stopping an inappropriate fear or aggressive response (Figure 4B). If the cortical EEG and facial recognition are matched—the system would lie dormant and allow for an appropriate fear or aggression response (Figure 4C). As our understanding of the underlying circuitry of ASD, schizophrenia, and behavior in general become more refined, other nodes may emerge as targets, either for stimulation or sensing.

## Conclusion

As technology advances in conjunction with our understanding of brain circuitry and neurotransmitter disorders, the opportunities to treat patients with heretofore treatment-resistant “behavioral” diseases are pregnant with promise. These patients with limited medical options may 1 day be able to break free from their behavioral and interpretive constraints. As history has shown, the treatment of these disorders is a challenging ethical field fraught with paternalism and loss of individual autonomy. Dissidents and proponents abound on each side of any argument—the resultant din can color or stall discussions that arise with every new advancement. CLDBS systems are on the horizon and the vanishing point may not be so distant; cautious progress should be our guide.

## Author contributions

RB this author is the primary author for the paper. J-PL senior author, editing, intellectual guidance, final approval.

### Conflict of interest statement

The authors declare that the research was conducted in the absence of any commercial or financial relationships that could be construed as a potential conflict of interest.
